# Quantification of unbound concentration of ticagrelor in plasma as a proof of mechanism biomarker of the reversal agent, MEDI2452

**DOI:** 10.1371/journal.pone.0201202

**Published:** 2018-07-26

**Authors:** Ann-Sofie Sandinge, Annika Janefeldt, Susanne Pehrsson, Sven Nylander

**Affiliations:** Cardiovascular, Renal and Metabolism, Innovative Medicines and Early Development Biotech Unit, AstraZeneca, Gothenburg, Sweden; Ehime University Graduate School of Medicine, JAPAN

## Abstract

Ticagrelor, a P2Y_12_ antagonist, is approved for prevention of thromboembolic events. MEDI2452 is a potential reversal agent for ticagrelor and ticagrelor active metabolite (TAM). The total plasma exposure of ticagrelor and TAM in patients are roughly 0.5–1 and 0.2–0.5 μmol/L, respectively. Both have similar high potency vs. P2Y_12_ (Ki 2 nmol/L) but are plasma protein-bound to 99.8% and only the 0.2% free fraction is able to inhibit the P2Y_12_ receptor. Thus, for unbound concentration measurements to be a proof of mechanism biomarker for MEDI2452 a very high sensitivity is required. Using established techniques as equilibrium dialysis and LC-MS/MS, made it possible to evaluate the efficacy of the reversal agent by measuring reduction of unbound concentration of ticagrelor in the presence of MEDI2452. With challenges such as ultra-low concentrations, small sample volumes, recovery issues and adsorption to plastic we managed to develop a highly sensitive assay for determining unbound concentration levels of ticagrelor and TAM in plasma with a quantification limit of 30 pmol/L and 45 pmol/L, respectively. With this method we were able to detect close to a 100-fold MEDI2452 mediated reduction in the unbound concentration of both ticagrelor and TAM. The assay provided proof of mechanism as MEDI2452 concentration- and dose-dependently eliminated unbound concentration of ticagrelor and reversed its antiplatelet activity in preclinical models and will support future development of MEDI2452.

## Introduction

Ticagrelor is an oral P2Y_12_ antagonist currently approved for the treatment of patients with acute coronary syndrome [1] or prior myocardial infarction [2].

Unlike the thienopyridine P2Y_12_ antagonists (clopidogrel and prasugrel), ticagrelor does not require metabolic activation. However, its main circulating metabolite, AR-C124910XX, has similar P2Y_12_ potency as parent ticagrelor and is here referred to as ticagrelor active metabolite (TAM). In patients, TAM has been shown to be present at 30–40% plasma exposure relative to the parent ticagrelor [3]. Thus, in order to effectively reverse the effect of ticagrelor treatment a reversal agent needs to bind up and neutralize both ticagrelor and TAM.

Increased risk of bleeding is a complication common for all antiplatelet therapies [1, 4, 5]. MEDI2452 is developed as a specific reversal agent for ticagrelor to be used in those rare patients that experience an uncontrolled emergency bleeding event or that need urgent surgery and cannot wait the 5–7 days wash out period [6].

MEDI2452 binds to circulating ticagrelor and TAM with an affinity of 20 pmol/L, which is 100-fold higher than the affinity of ticagrelor for the P2Y_12_ receptor (2 nmol/L [7]). The antigen-binding fragment was isolated from a human antibody phage library by selection on biotinylated ticagrelor, followed by screening for specificity [8].

MEDI2452 binds to the protein unbound concentration (*Cu*) of ticagrelor and TAM in plasma and is not expected to bind to ticagrelor bound to plasma proteins [8]. By removing the unbound concentration MEDI2452 will restore platelet function as it is only unbound ticagrelor that can bind to and inhibit P2Y12. As the plasma protein binding of ticagrelor and TAM is high (>99.8% [9]) the fraction unbound (*fu*) is only going to be about 0.2%. The mean maximal total (free and protein bound) plasma concentrations of ticagrelor and TAM in patients following 4 weeks of ticagrelor treatment (90 mg bid) have been documented to be 1.5 and 0.5 μmol/L (770 and 257 ng/mL [9]), respectively. This corresponds to an expected unbound concentration of ticagrelor and TAM of 4.4 nmol/L and 1.6 nmol/L, respectively. Previous data, using the assay here described, support that the unbound plasma concentration of both ticagrelor and TAM need to be reduced below 0.05 nmol/L to completely restore platelet function [8]. As MEDI2452 bind up and neutralize unbound ticagrelor more unbound ticagrelor is dissociated from plasma proteins and made available for MEDI2452. Due to the much higher affinity, 20 pM, of MEDI2452 versus ticagrelor compared to the unspecific interaction between ticagrelor and plasma proteins the equilibrium favors MEDI2452-bound ticagrelor. The expected half-life of MEDI2452 is about 12h which is similar to that of ticagrelor and TAM in humans, which is 9.8 and 12.4 hours, respectively [8]. Thus, it should be feasible to restart ticagrelor treatment soon thereafter. To enable detection of levels corresponding to no effect limit in terms of inhibition of platelet function (predicted to 0.05 nmol/L), a highly sensitive assay is required. Consequently, we set out to develop an analytical method for ticagrelor and TAM with a lower limit of quantification (LLOQ) of 0.03 nmol/L to accurately determine the unbound concentrations of ticagrelor and TAM after MEDI2452 treatment to support proof of mechanism.

There are two predominant techniques for studying the unbound concentration in plasma, equilibrium dialysis (ED) and ultrafiltration (UF) [10]. Previous evaluation of the fraction unbound for ticagrelor and TAM using UF methodology, indicate that this technique is unsuitable due to the high non-specific adsorption (NSA) [11] why ED was chosen for further evaluation. The plastic material used in ED is subject for compound binding but the biggest advantage with this method is that NSA is believed not to affect the fraction unbound since the system is in equilibrium [10].

In ED, two aliquots are separated by a semipermeable membrane. Plasma sample is placed on one side of the membrane and a phosphate buffer on the corresponding side. Semipermeable membranes with different cut-offs can be selected depending on the size of the molecule to be separated and an equilibrium will normally be established after 4–24 hours. The equilibrium can be affected by changes in pH, temperature, drug concentration, protein concentration and the concentration of other drugs present [10].

Here we present the development and optimization of a highly sensitive assay enabling detection of MEDI2452-mediated concentration- and dose-dependent elimination of unbound concentrations of ticagrelor and TAM. The sensitivity of the assay, 0.03 nmol/L, was substantially enhanced, compared with previous reported LC-MS/MS methods with LLOQ of 5 ng/mL (~10 nmol/L) [11, 12]. Sample volume for ED has been optimized taken in consideration limitation in sample volume from mice studies. Recovery after preparation of total plasma (undialyzed plasma) and plasma retentate (dialyzed plasma) in samples with or without the reversal agent has been evaluated as well as possible concentration dependency in measurements of the fraction unbound of ticagrelor due to NSA.

This highly sensitive assay for unbound concentrations of ticagrelor and TAM will be an important tool in the future development of MEDI2452.

The aim of this work was to set up a method for measuring unbound concentrations of ticagrelor and TAM in presence of MEDI2452 and not designed to prove efficacy. The work was exploratory in nature and therefor components for methodological rigor were not included in the experimental design [13].

## Materials and methods

### Chemicals and materials

Ticagrelor (mw 522.57 g/mol), TAM (AR-C124910XX, mw 478.52 g/mol) and the internal standard (D_7_-ZD6140) were from AstraZeneca R&D (Gothenburg, Sweden) and MEDI2452 (mw 50 kDa) from MedImmune (Cambridge, UK). Acetonitrile, Optima^®^ LC/MS grade, was purchased from Fisher Scientific UK (Loughborough, UK), purified water was obtained from ELGA Purelab Ultra (Glostrup, Denmark) and Milli-Q^®^ water from Merck Millipore (Solna, Sweden). Ammonium acetate, acetic acid and sodium phosphate were purchased from Merck (Darmstadt, Germany), sodium chloride from Acros Organics (New Jersey, USA) and sodium azide from Sigma-Aldrich (St Louis, MO, USA). Eppendorf^®^ Protein LoBind tubes 1.5 mL (Cat No 022431081) and Eppendorf^®^ LoBind 96-deepwell plates, 0.5 mL (Cat No 951032107) were used. 96-well DispoEquilibrium DIALYZER Plates, MWCO 5000 Dalton (Cat No SP-742330), and Plate Rotator Dual (Cat No SP-742334) were purchased from CMA Microdialysis AB (Kista, Sweden). The plates were shaken using a multi tube vortex VX-2500 from VWR and protein precipitation was performed using a 96-hand Bravo^TM^ Velocity11^TM^ robot from Agilent Technologies.

### Human samples

Informed consent was obtained from all subjects, and the study was performed in accordance with local ethical regulations and was approved by the Research Ethics Board of Gothenburg, Sweden. All subjects abstained from drugs known to affect platelet function for at least 10 days. Blood was collected from seven fasted healthy female caucasian volunteers by vein puncture of vena cephalica. The first 2 mL of blood was discarded prior to collecting aliquots into tubes containing 0.109 mol/L sodium citrate, 1+9 (citrate + blood), to a final concentration of 10.9 mmol/L. The anticoagulated human blood was centrifuged at 240 x g for 15 min and platelet-rich plasma (PRP) was carefully removed and transferred to a clean vial.

Human platelet aggregation was studied using light transmission aggregometry as described previously [8]. Briefly, PRP was pre-incubated with 1 μmol/L ticagrelor for 1 hour before co-incubation with MEDI2452 for 30 minutes, followed by initiation of aggregation by 20 μmol/L ADP. Parallel samples of PRP collected after 30 minutes of co-incubation with MEDI2452, but before ADP-induced aggregation, was used for analysis of unbound concentrations of ticagrelor in plasma.

### Mouse samples

All experiments were performed in accordance with relevant guidelines and regulations, the animal work was approved by the Local Animal Research Ethics Board of Gothenburg, Sweden. As described previously [14], mice were pretreated with a bolus dose of ticagrelor of 240 μg/kg/min over 5 minutes, followed by a continuous infusion of 30 μg/kg/min for 15 minutes, before a bolus dose of 50–250 mg/kg MEDI2452 was given over 45 seconds. Terminal blood samples for platelet aggregation and analysis of unbound concentration of ticagrelor in plasma were collected 30 minutes after end of ticagrelor infusion. Platelet aggregation was studied using ADP-induced whole blood platelet aggregation as described previously [14].

### Pig samples

The study was performed in accordance with relevant guidelines and regulations, the animal work was approved by the Local Animal Research Ethics Board of Gothenburg, Sweden. As described previously [15], three groups of Swedish landrace pigs were given either ASA (acetylsalicylic acid) alone, ASA + ticagrelor or ASA + ticagrelor + MEDI2452.

250 mg ASA was given orally two days prior to experiment and in addition an i.v. injection of 10 mg kg^-1^ was given on experimental day. Ticagrelor, 3 mg kg^-1^, or vehicle, was administrated i.v. for 60 minutes. An acute major bleed was caused by having a piece of the central liver lobe cut off immediately followed by administration of MEDI2452, 600 mg kg^-1^, or vehicle, for 5 minutes. Arterial blood was collected at baseline after administration of ASA but before ticagrelor, and after the end of infusion of ticagrelor or vehicle. In addition, blood was collected at ten time-points (5–240 minutes) after administration of MEDI2452 or vehicle.

### Equilibrium dialysis

The 96-well dialysis plates were prepared by addition of 100–190 μL dialysis buffer (42.2 mM NaH_2_PO_4_, 79.2 mM Na_2_HPO_4_, 75 mM NaCl and 30 mM NaN_3_ (final concentrations), pH 7.0 in ELGA H_2_O) per well to the buffer side of the plate. The wells were sealed and the plate was left to soak for 10–15 minutes. Plasma samples were thawed at room temperature and vortex mixed before addition of an equal volume of plasma sample to the opposite well. The wells were sealed and the plate was placed in a Plate Rotator in a 37°C incubator. Dialysis time was set to 24 hours according to previous evaluations [11]. Stability of ticagrelor and TAM in plasma and stability of the partitioning between protein bound and unbound fractions were assessed and evaluated in previous studies [11].

### Plasma retentate sample preparation

Retentate, 50 μL, from the plasma side of the 96-well dialysis plate was transferred to a 96-well LoBind PCR clean eppendorf plate. Prior to protein precipitation 10 μL of either 1% or 10% formic acid was added to the sample to a final concentration of 0.17% or 1.7%, respectively, directly followed by vortex mix (10 minutes). Thereafter 180 μL 100 nmol/L internal standard in acetonitrile was added to each sample directly followed by vortex mix (10 minutes) and centrifugation (3220 g, 20 minutes, 4°C). Supernatant, 75 μL, was transferred to a new plate and diluted 1:1 with purified water. The samples were vortex mixed and analyzed by LC-MS/MS.

### Plasma dialysate sample preparation

Dialysate, 50 μL, from the buffer side of the 96 -well dialysis plate was transferred to a 96-well LoBind PCR clean eppendorf plate containing 50 μL 100 nmol/L internal standard in acetonitrile. The tips were rinsed by pipetting up and down two to three times. The samples were vortex mixed and analyzed by LC-MS/MS.

### Calculations

The fraction unbound, *fu*, is calculated according to the following equation;

$$fu=\frac{Cb\times Db}{Cp\times Dp}$$

D denotes the dilution factor and the sub-fixes *Cb* and *Cp* represent concentrations in buffer and plasma samples, respectively.

The unbound concentration, *Cu*, is calculated according to the following equation;

Cu=fu×Ctot

*Ctot* denotes the total plasma concentration (*Cu* + protein bound concentration).

### LC-MS/MS analysis

Chromatographic separation was performed using an ACQUITY UPLC^®^ I-class system (Waters Corporation, Milford, Massachusetts, USA). An analytical column, ACQUITY UPLC^®^ BEH C18, 2.1x50 mm column, was used with a 1.7 μm particle size maintained at 40°C. Mobile phase A consisted of Milli-Q water with 2% acetonitrile and 10 mmol/L ammonium acetate, pH 5, and mobile phase B consisted of acetonitrile with 10 mmol/L ammonium acetate, pH 5. Sample injection volume was 1–5 μL and the analytical run was 4% B at t_0_, ramp to 95% B from t_0_ to t_1.5_ followed by a 0.8 minutes pause before returning to 4% B at a constant flow rate of 0.7 mL/min. The retention time for ticagrelor, TAM and internal standard was approximately 1.22 minutes.

The mass detector was a Waters Xevo^TM^ TQ-S triple quadrupole mass spectrometer (Waters Corporation, Milford, MA, USA) using electrospray ionization. The mass spectrometer was operated in multiple reaction monitoring mode (MRM). Samples were ionized in negative mode and the ion source was set to a temperature of 150°C. Ionization source parameters were as follows: capillary voltage 0.60 kV, cone 60 V, source offset 60 V. Gas settings were as follows: cone gas 150 L/h, desolvation gas 1200 L/h and nebulizer gas flow 7 bar. Dwell time per transition was set to 0.050 seconds. Resolution settings were for low mass 2.7 and for high mass 14.6 for both Q1 and Q3. The following MRM transitions were monitored with collision energies given in parenthesis: ticagrelor *m/z* 521.11 → 360.99 (22 V); TAM *m/z* 477.27 → 361.07 (22 V); internal standard D_7_-XD6140 *m/z* 528.37 → 368.22 (28 V). Data was processed using TargetLynx^TM^ software (Waters Corporation, Milford, MA, USA). A calibration curve was generated for both ticagrelor and TAM in both plasma and buffer matrix, by plotting nominal concentrations of calibration standards versus the analyte to internal standard peak area ratio. Sample concentrations were calculated by linear regression analysis (y = ax + c), using the reciprocal of concentration (1/x) as weighting.

### Calibration standards and internal standard preparation

Stock solutions of ticagrelor, TAM and internal standard were prepared separately with a concentration of 1000 μmol/L in acetonitrile/water (90/10) and stored at 4°C. Calibration standard samples of ticagrelor and TAM were freshly prepared by serial dilution in 50% acetonitrile at eight concentrations in a range from 20000 nmol/L to 0.3 nmol/L in 1.5 mL LoBind eppendorf tubes. For non-dialyzed plasma (total concentration) and ED retentate analysis, the calibration standard samples of the six highest concentrations were further diluted, 10 times, in blank plasma matrix to produce standards for ticagrelor of 2000, 250, 50, 10, 2 and 0.4 nmol/L and standards for TAM of 1500, 375, 75, 15, 3 and 0.6 nmol/L. For ED dialysate analysis, the calibration standard samples of the six lowest concentrations were further diluted, 10 times, in PBS pH 7.4:acetonitrile (50:50, v/v), containing internal standard (100 nmol/L). The final calibration concentrations for dialysate analysis were 50, 10, 2, 0.4, 0.06 and 0.03 nmol/L for ticagrelor standards and 75, 15, 3, 0.6, 0.1 and 0.045 nmol/L for TAM standards. A stable isotope labeled internal standard (D_7_-ZD6140) was used to quantify the area response of ticagrelor and TAM. A stock solution of 1000 μmol/L D_7_-ZD6140 in 90% acetonitrile was further diluted with 100% acetonitrile to a concentration of 100 nmol/ L and stored at 4°C for retentate and dialysate analysis.

## Statistics

The results are presented as the mean ± standard deviation (SD). Statistical analyses were performed using GraphPad prism version 7.02. Multiple comparisons in the dialysis sample volume and the NSA experiments were performed using one-way ANOVA with Tukey’s multiple comparisons test. For comparisons of formic acid addition in plasma retentate a two-way ANOVA with Sidak’s multiple comparison test was used. Values of p < 0.05 was considered statistically significant.

## Results

### Equilibrium dialysis and sample preparation

**Fig 1:**
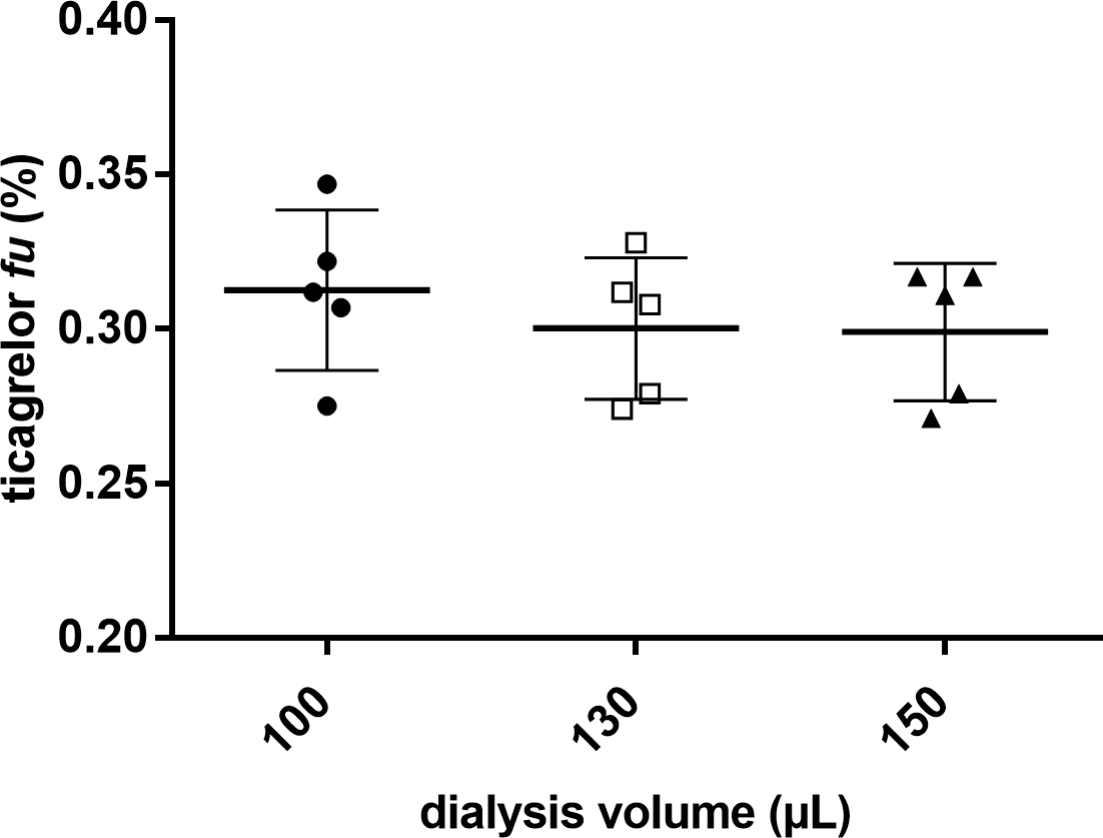
Reproducibility of ticagrelor fraction unbound (*fu*) at different plasma volumes in the dialysis. Human plasma samples were incubated with ticagrelor, 1 μmol/L (theoretical *fu* value ~ 0.2%). Data expressed as mean ± SD (n = 5). Statistical differences were calculated using one-way ANOVA test (p = 0.619). Tukey’s multiple comparison test were used to look at differences between the volume groups, 100 μL vs. 130 μL (p = 0.692), 100 μL vs. 150 μL (p = 0.649).

To optimize the equilibrium dialysis method, experiments to assess precision of volumes in the dialysis plate were performed. Human plasma was spiked with ticagrelor and three separate sample volumes (100, 130 and 150 μL) were subjected to ED and LC-MS/MS analysis in five replicates. The SD of the calculated *fu* within each volume group was 7.81 ± 0.53% whereas between the groups it was 2.48% (Fig 1). Statistical analyses show no significant differences between the three sample volumes.

**Fig 2:**
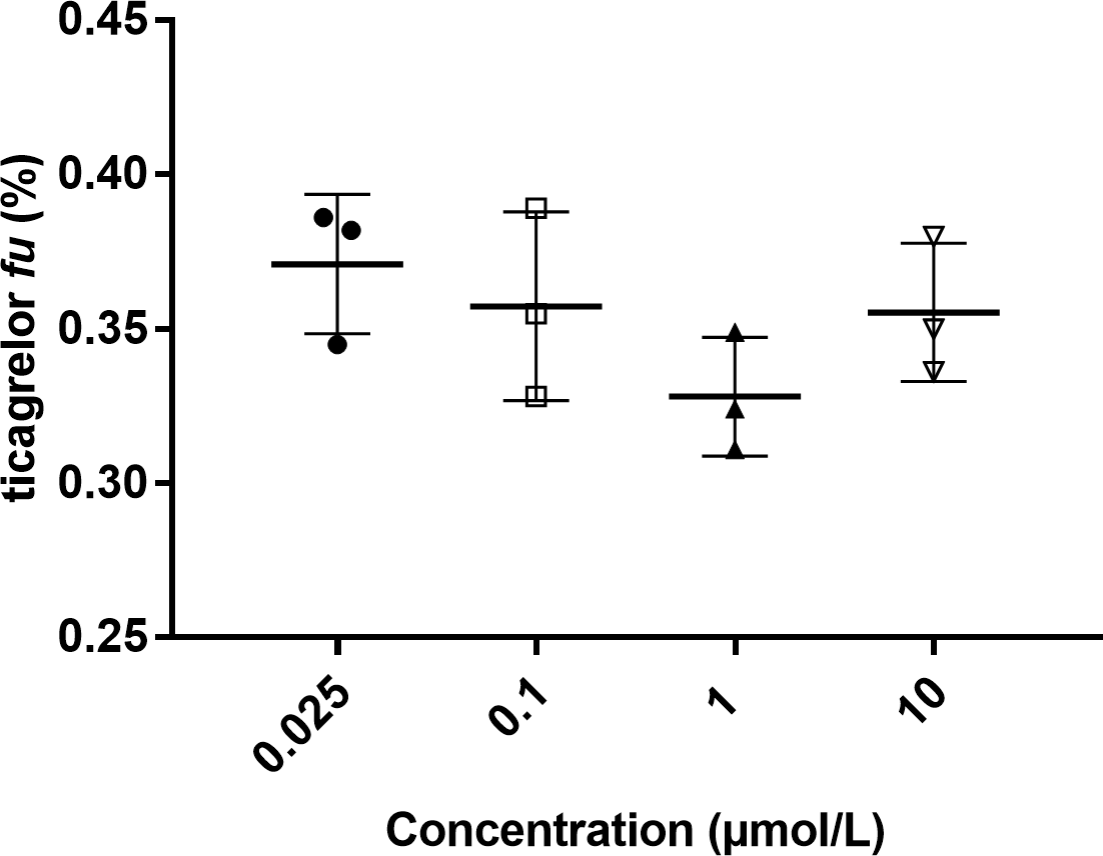
Ticagrelor fraction unbound (*fu*) measured in plasma samples with different concentrations of ticagrelor. Human plasma sample pre-incubated in vitro for 60 minutes. Data expressed as mean ± SD (n = 3). Statistical differences were calculated using one-way ANOVA test (p = 0.249). Tukey’s multiple comparison test was used to look at differences between the lowest, 0.025 μmol/L, and highest, 10 μmol/L, concentration groups (p = 0.845).

To study the effect of NSA during ED, the precision of determination of the unbound fraction in plasma samples pre-incubated (60 minutes) with different concentrations of ticagrelor was evaluated. Three replicates of 100 μL human plasma samples pre-incubated with one of four different ticagrelor concentrations (0.025–10 μmol/L) were subjected to ED followed by LC-MS/MS analysis. The SD was 6.75 ± 2.25% within the groups and 5% between the groups (Fig 2). Statistical analyses show no significant differences between the four concentration levels.

The extent of adsorption to plastic in ED-buffer during sample preparation, was investigated. ED-buffer and ED-buffer:acetonitrile (1:1) were pre-incubated with 0.1 μmol/L ticagrelor and transferred to a generic Safe-Lock tube or a LoBind tube. Samples were immediately transferred in six steps to empty tubes, one sequence for each tube type. Recovery, ratio between peak area from tube 6 and tube 1, after the transfer experiment with ED-buffer in generic Safe-Lock tubes was 38.0 ± 11.9% compared with 73.0 ± 1.8% in LoBind tubes. Recovery after the experiment with ED-buffer:acetonitrile (1:1) in both generic Safe-Lock and LoBind tubes was 100% (Table 1).

**Table 1:** Comparison of recovery using LoBind tubes and generic Safe-Lock tubes.

Tube (solution)	Recovery (area tube 6/area tube 1)
Generic (ED buffer)	38.0 ± 11.9%
Generic (ED buffer: acetonitrile)	101 ± 1.2%
LoBind (ED buffer)	73.0 ± 1.8%
LoBind (ED buffer: acetonitrile)	102 ± 1.8%

Recovery after five transfer steps of 0.1 μmol/L ticagrelor diluted in either ED buffer or ED buffer:acetonitrile (1:1) in both LoBind tubes and generic Safe-Lock tubes. Data expressed as mean ± SD (n = 2).

**Fig 3:**
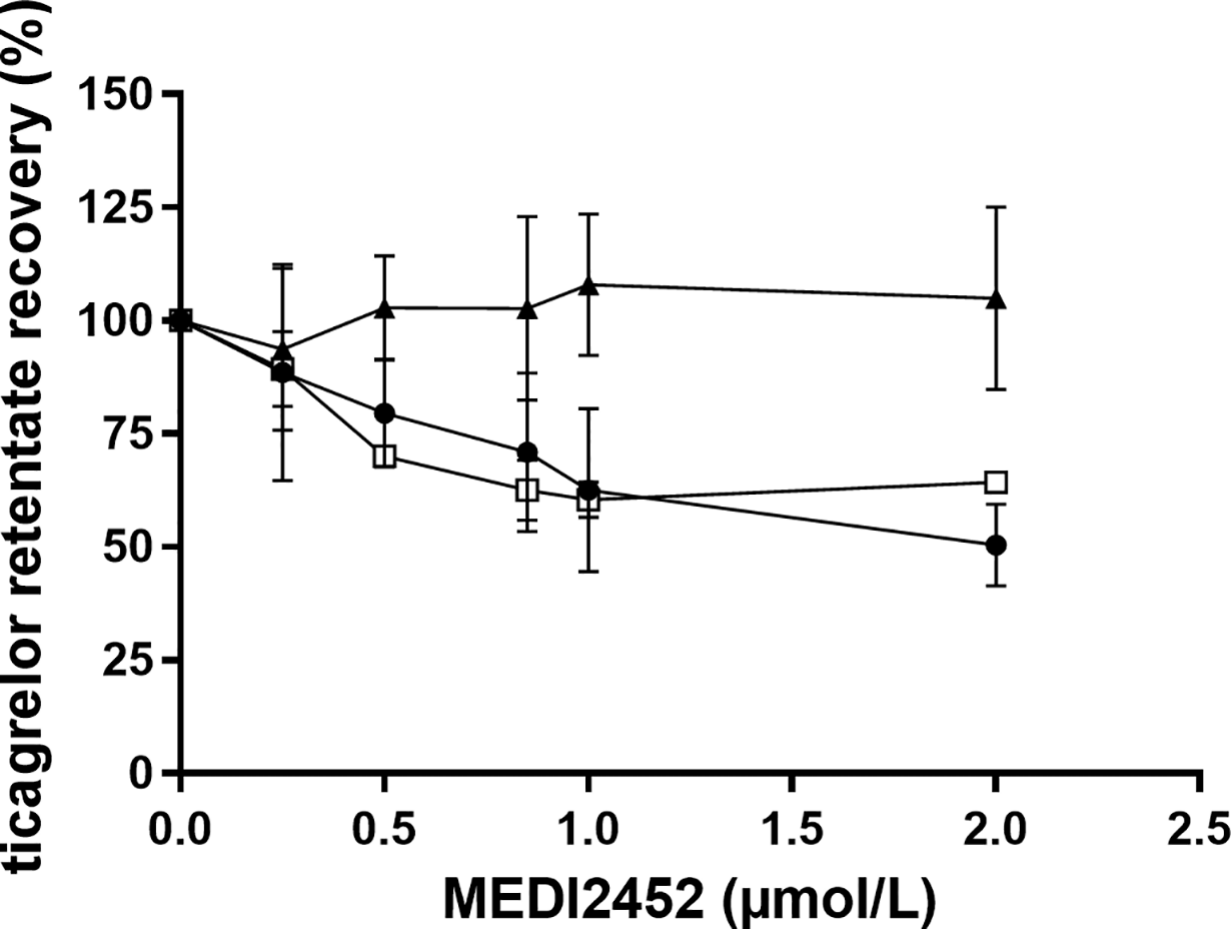
Recovery of ticagrelor in presence of MEDI2452 using different protocols to prepare the plasma retentate. No formic acid (●, n = 4), 0.17% formic acid (final concentration) added at time of protein precipitation (□, n = 2) and 0.17% formic acid (final concentration) added 10 minutes prior to protein precipitation (▲, n = 8). Human PRP was pre-incubated in vitro with ticagrelor (1 μmol/L) for 60 minutes followed by addition of MEDI2452 (0.25, 0.5, 0.75, 1 and 2 μmol/L) and 30 minutes further incubation. Data expressed as mean ± SD.

Recovery of ticagrelor in the presence of MEDI2452 (Ticagrelor:MEDI2452 1:1) after sample preparation of plasma retentate was evaluated. Formic acid was added to a final concentration of 0.17%, to the plasma retentate followed by vortex for 10 minutes prior to protein precipitation. The samples were compared with plasma retentate with no addition of formic acid or addition of formic acid by the time of protein precipitation. The addition of formic acid prior to protein precipitation at MEDI2452 concentration of 2 μmol/L gave a recovery of 105 ± 20% compared with 50.5 ± 9.0% with no addition of formic acid or 64.3 ± 1.1% with the addition of formic acid at time of protein precipitation (Fig 3).

**Fig 4:**
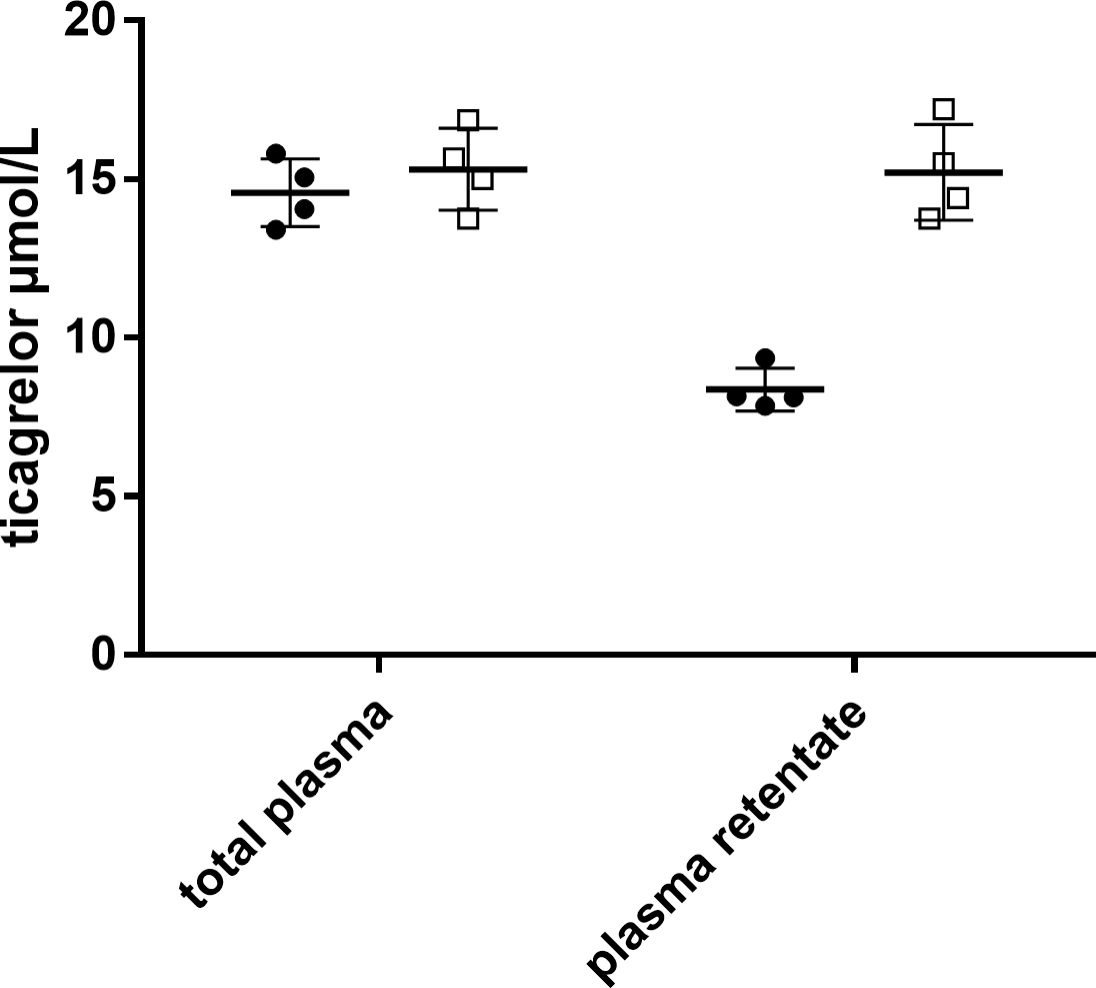
Ticagrelor concentrations in non-dialyzed plasma and plasma retentate after addition of formic acid. Addition of 0.17% or 1.7% formic acid (final concentrations) to pig plasma samples were made 10 minutes prior to protein precipitation. Aspirin pre-treated pigs were infused with ticagrelor (3 mg/kg/h) for 60 minutes followed by a MEDI2452 bolus (600 mg/kg) [15]. Samples were collected 15 minutes after MEDI2452 bolus. Data expressed as mean ± SD (n = 4). Statistical differences were calculated using two-way ANOVA test (p = 0.0022). Sidak’s multiple comparison test was used to look at differences between plasma retentate with 0.17% formic acid compared with plasma retentate with 1.7% formic acid (p = 0.0008).

To evaluate sample preparation further, four in vivo plasma samples from a study in pigs [15] dosed with ticagrelor and MEDI2452 were subjected to ED. After sample collection, formic acid was added to the final concentration of either 0.17% or 1.7% to retentate sample prior to protein precipitation. The ticagrelor concentrations in retentate plasma were compared with the ticagrelor concentrations in non-dialyzed plasma samples that had been subjected to the same sample preparation procedure as the plasma retentate. Samples that had not been subjected to ED showed no differences in concentrations when treated with 0.17% or 1.7% formic acid, final concentrations, prior to protein precipitation. However, in samples that had been dialyzed over night the concentration of ticagrelor in plasma retentate treated with 0.17% formic acid prior to protein precipitation was 8.37 ± 0.68 μmol/L, compared with 15.2 ± 1.52 μmol/L in samples treated with 1.17% formic acid prior protein precipitation (Fig 4). Statistical analyses show significant differences between plasma retentate treated with 0.17% formic acid compared with the other variables (p = 0.0022).

### LC-MS/MS analysis of ticagrelor and TAM

**Fig 5:**
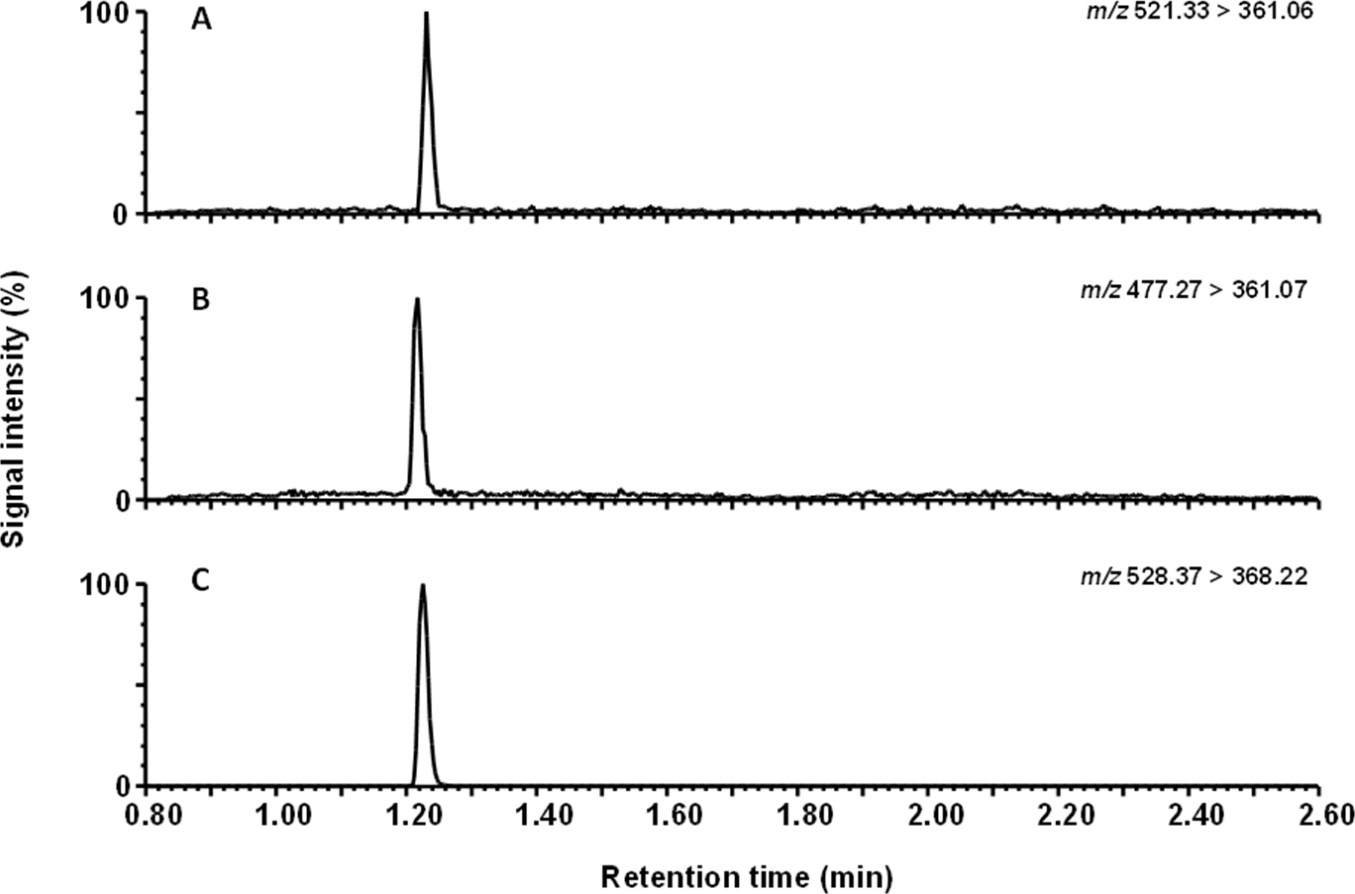
Representative LC-MS/MS chromatograms of calibration samples in buffer. (A) Ticagrelor, 0.4 nmol/L (B) TAM, 0.6 nmol/L and (C) internal standard.

**Fig 6:**
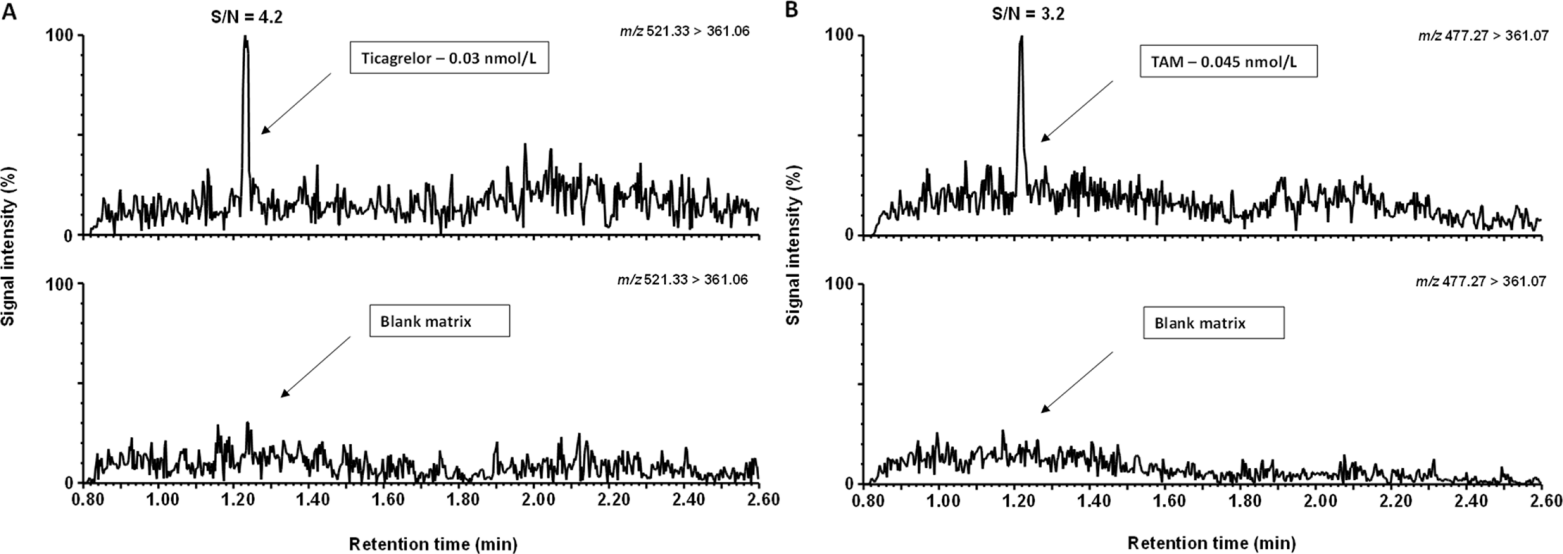
Representative chromatograms displaying the signal to noise ratio at the lowest calibration levels. (A) Ticagrelor, 0.03 nmol/L and (B) TAM, 0.045 nmol/L.

**Fig 7:**
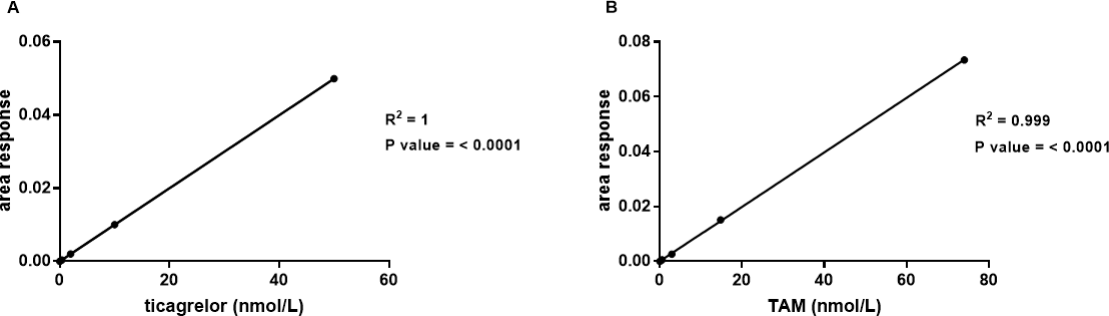
Representative calibration curves for ticagrelor and TAM. (A) Ticagrelor, 0.030–50 nmol/L and (B) TAM, 0.045–75 nmol/L. Linear regression was used. Data expressed as mean ± SD (n = 3).

Typical extracted-ion chromatograms for ED-buffer:acetonitrile 1:1 calibration samples of ticagrelor, TAM and ticagrelor internal standard are shown in Fig 5. After signal to noise determination, the LLOQs were 0.03 and 0.045 nmol/L for ticagrelor and TAM, respectively (Fig 6). Based on the signal to noise assessment and the precision at the LLOQ (≤ 20%) we could quantify the unbound concentration of ticagrelor and TAM present in plasma dialysate down to their respective LLOQs. The ED-buffer:acetonitile (1:1) calibration curve was linear from 0.03 to 50 nmol/L for ticagrelor and 0.045 to 75 nmol/L for TAM (Fig 7).

### Reduction of the unbound concentration of ticagrelor predicts the reversal of ADP-induced human platelet aggregation, in vitro

**Fig 8:**
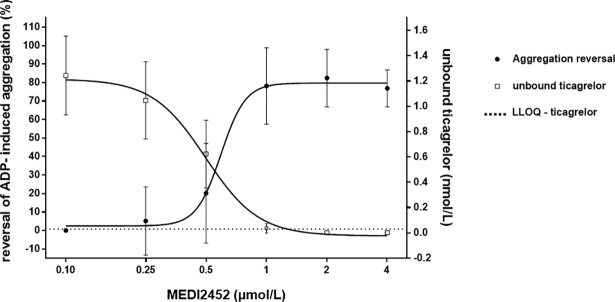
MEDI2452 mediated reduction of the unbound concentration of ticagrelor in human plasma and corresponding reversal of platelet aggregation. Human plasma samples were pre-incubated in vitro with 1 μmol/L ticagrelor prior to addition of MEDI2452. Data expressed as mean ± SD of five separate experiments and replotted from previously reported data [8].

As previously published, maximal anti-platelet reversal in 1 μmol/L ticagrelor pre-incubated blood samples was achieved at 1 μmol/L MEDI2452, given that MEDI2452 binds 1:1 to ticagrelor [8]. At a concentration of 1 μmol/L MEDI2452, the unbound plasma concentration of ticagrelor had decreased from 1.2 nmol/L to <0.05 nmol/L (Fig 8).

### Reduction of the unbound concentration of ticagrelor and TAM predicts the reversal of ADP-induced aggregation, in vivo

**Fig 9:**
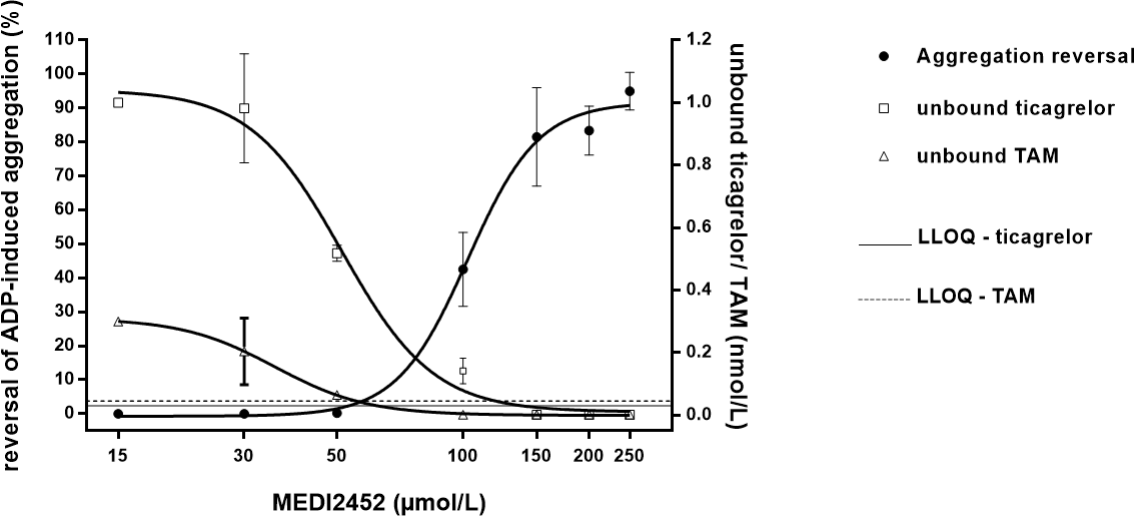
MEDI2452 mediated reduction of the unbound concentration of ticagrelor and TAM in mouse plasma and corresponding reversal of platelet aggregation. MEDI2452 was dosed in vivo to ticagrelor treated mice. An intravenous bolus of MEDI2452 (50, 100, 150, 200, 250 mg/kg) was given just after termination of intravenous ticagrelor infusion (1.2 mg/kg bolus + 30 μg/kg*min) and blood samples were collected 30 minutes after MEDI2452 dosing. Data expressed as mean ± SD of 2–6 separate experiments and replotted from previously reported data [14].

Similar to the in vitro conditions, reversal of platelet aggregation is perfectly predicted by reduction of the unbound plasma concentrations of ticagrelor and TAM after dosing MEDI2452 (50, 100, 150, 200, 250 mg/kg) in vivo to ticagrelor treated mice (Fig 9).

## Discussion

For the purpose to evaluate the efficacy of the ticagrelor reversal agent, MEDI2452, we have refined and optimized a methodology, using a combination of equilibrium dialysis and LC-MS/MS technology, to detect unbound concentrations of ticagrelor and TAM in plasma down to 0.03 and 0.045 nmol/L, respectively. We have also optimized the sample preparation of non-dialyzed plasma and plasma retentate (both representing the total plasma concentrations), compared with previous published method [11] in samples containing MEDI2452. The high degree of protein binding for both ticagrelor and TAM (99.8%) made the sensitivity of the assay crucial. The mean maximal therapeutic levels after 4 weeks of ticagrelor treatment (90 mg twice daily) ticagrelor and TAM have been documented as 1.5 and 0.5 μmol/L, respectively [3]. The corresponding mean maximal unbound concentrations of ticagrelor and TAM are <64 nmol/L and <1.6 nmol/L, respectively. Thus, to accurately detect the decrease of the unbound concentrations after MEDI2452 treatment a highly sensitive triple quadrupole instrument was essential. With this method we were able to detect close to a 100-fold MEDI2452 dose- and concentration-dependent reduction in the unbound concentration of both ticagrelor and TAM. Ticagrelor and TAM are hydrophobic molecules and binds to plastic in aqueous environments. Adsorption to plastic materials was minimized by the use of low binding materials and the addition of acetonitrile into tubes and wells prior to sample transfer and dilution. The degree of NSA during the 24 hours of ED was considered to be controlled since the level of the fraction unbound gave comparable results at four different concentrations, 0.025–10 μmol/L. Mouse experiment only provide small plasma sample volumes why evaluation of the robustness of the ED method with the existing volumes was important. The sample volume range for the dialysis plates were 50–300 μL according to product description. Sample volumes below 100 μL were difficult to handle and gave low reproducibility while sample volumes ≥100 μL gave high reproducibility and good precision why 100 μL was set to be the minimal sample volume. The recovery of ticagrelor and TAM in non-dialyzed plasma and plasma retentate in samples containing MEDI2452 (1:1) was a key element for success and the addition of formic acid prior to protein precipitation increased the recovery dramatically from 60% to 100%. The formic acid appears to act as solvent to facilitate the break of bonds between ticagrelor and MEDI2452 and thereby increase the recovery of ticagrelor and TAM during the protein precipitation. We found that the formic acid level needed to be higher after ED compared to non-dialyzed plasma samples possibly due to conformational changes of the reversal agent-ticagrelor/TAM-complex during the 24 hours of ED. Highly sensitive and accurate detection of the unbound concentrations of ticagrelor and TAM was key for the pre-clinical evaluation of MEDI2452 as previously reported [6]. Maximal reversal of ADP-induced human platelet aggregation in the presence of 1 μmol/L ticagrelor in vitro, was achieved at 1 μmol/L MEDI2452 supporting that MEDI2452 binds 1:1 to ticagrelor [8]. At 1 μmol/L MEDI2452, the unbound concentration of ticagrelor in plasma decreased from 1.2 nmol/L to <0.05 nmol/L. These in vitro data translated to in vivo as a very similar profile of reduction of the unbound concentrations paralleled recovery of ADP-induced platelet aggregation in mice dosed with ticagrelor and MEDI2452 [14]. Thus, using the described analytical methodology to accurately measure sub-nanomolar levels of ticagrelor and TAM, both proof of mechanism and proof of concept for MEDI2452 was achieved—elimination of free ticagrelor and parallel restoration of platelet aggregation.

## Conclusions

Optimization of volume, sample handling for avoiding adsorption to plastic material, sample preparation and LC-MS/MS methodology, allowed us to achieve the impressive LLOQ of 0.03 nmol/L. At concentration ratio 1:1, ticagrelor: MEDI2452, ADP-induced aggregation was restored and the unbound concentration of ticagrelor in plasma was decreased from 1.2 nmol/L to <0.05 nmol/ L. Thus, using the described assay enabled definition of the PKPD relationship between MEDI2452, its proof of mechanism biomarker (unbound concentration of ticagrelor), and the ticagrelor biomarker (ADP-induced platelet aggregation). The assay supported non-clinical proof of concept for MEDI2452 and will support the future clinical development of MEDI2452.

## Supporting information

S1 File Relevant data underlying the results described in manuscript. (XLSX)

## Abbreviations

ADP: adenosinE diphosphate
ASA: ACETYLSALICYLIC ACID
ED: Equilibrium Dialysis
FU: fraction unbound
LLOQ: LOWER LIMIT OF QUANTIFICATION
MRM: Multiple Reaction Monitoring
MWCO: molecular weight cut off
NSA: NON- SPECIFIC ADSORPTION
PRP: PLATELET-rich plasma
TAM: ticagrelor active metabolite
UF: Ultra filtration
